# Wisely frugal: ensuring sustainable funding for novel cancer therapeutics through a budget impact analysis in resource-limited settings

**DOI:** 10.3332/ecancer.2025.1941

**Published:** 2025-07-02

**Authors:** Nuradh Joseph, Vimukthini Peiris, Vodathi Bamunuarachchi, Prasad Abeysinghe, Nadarajah Jeyakumaran, Devinda Jayathilake, Kanthi Perera, Rohini Fernandopulle, Sanjeeva Gunasekera

**Affiliations:** 1 District General Hospital – Hambantota 82000, Sri Lanka; 2 Sri Lanka Cancer Research Group, Maharagama 10280, Sri Lanka; 3 District General Hospital – Vavuniya 43000, Sri Lanka; 4 Apeksha Hospital, Maharagama 10280, Sri Lanka; 5 Sir John Kotalawela Defence University 10390, Kandawala, Sri Lanka

**Keywords:** Essential drugs, medical oncology, cost-effectiveness, lower middle-income countries, novel drugs, cancer therapeutics, budget-impact analysis

## Abstract

**Introduction:**

Cancer care in Sri Lanka is predominantly provided through its state health system which is free at the point of delivery. We performed a budget impact analysis of novel cancer drugs with a view to enabling better prioritising of their procurement.

**Methods:**

Median survival gain was obtained for each indication of a novel cancer drug by a review of the literature. The direct cost of drug procurement was obtained from the Ministry of Health of Sri Lanka and the cost per life year gained was computed for each indication. Two thresholds - per capita gross domestic product (GDP) per life year gained (GDP × 1 = US$3815) and three times per capita GDP per life year gained (GDP × 3 = US$11445) were considered to determine cost effectiveness. The cumulative annual cost of these treatments was subsequently determined.

**Results:**

Data obtained on 42 novel cancer drugs spanning across 90 indications were included in the analysis. The cumulative annual treatment cost when the threshold was set at GDP × 1 was United States Dollar (US$) 6 million and it increased to US$ 13.2 million if the threshold was expanded (GDP × 3 = US$11445). Only 27 indications met the (GDP × 3 = US$11445) threshold while there were 18 drugs that did not meet the thresholds for any indication. Without a threshold, if every eligible patient were to receive treatment as currently indicated, the total cost would reach almost US$ 300 million per year.

**Conclusion:**

Budget impact analyses and defining cost-effectiveness thresholds will lead to considerable savings and help prioritise the procurement of novel agents in the state health system in Sri Lanka.

## Introduction

Sri Lanka has an age-adjusted annual incidence of 129 cancer cases per 100,000 population with nearly 32,000 new patients being diagnosed each year, according to data from the national cancer registry [1]. However, the actual incidence might be considerably higher due to under-reporting of cases [2].

Cancer care in Sri Lanka is predominantly provided through its public funded state health system which is free at the point of delivery^2,3^. Funded by general taxation, this system functions as a network of primary, secondary and tertiary care hospitals under the administrative control of the Ministry of Health [2,3]. Clinical oncology services are provided by 26 cancer centres located throughout the island^2,3^.

Each year, the Ministry of Health of the government of Sri Lanka allocates around 210 million United States Dollar (US$) for the procurement of drugs for hospitals under its purview [4]. At the beginning of 2022, Sri Lanka faced a foreign exchange crisis arising from years of imprudent macroeconomic fiscal policies aggravated by the Covid-19 pandemic [5].

The crisis was devastating and almost led to a calamitous breakdown of Health services, and procurement of pharmaceuticals from abroad proved extremely challenging^5^. Since virtually all oncology drugs are imported, the economic crisis posed a major threat to cancer care.

We performed a budget impact analysis of novel cancer drugs with a view to compiling a list of essential drugs and approved indications to help prioritise procurement and help mitigate the impact on cancer care.

## Methods

Our primary objective was to determine the treatment cost per life year gained for each indication of a novel cancer drug. Since the focus is on novel therapeutics, conventional anti-neoplastic agents where the total cost of treatment was less than 1,000 US$ were excluded from the analysis.

### Computing survival gain

Since data on quality-adjusted life years was not available in the local setting we considered life years gained as the outcome parameter. This was obtained from publications of pivotal randomised controlled trials for drug and indication.

In the palliative setting, median overall survival (OS) was the outcome variable. In trials, where there was a significant crossover of treatment, progression-free survival gain was considered, especially if reliable estimates of OS were not available.

**Supplementary Figure S1.**
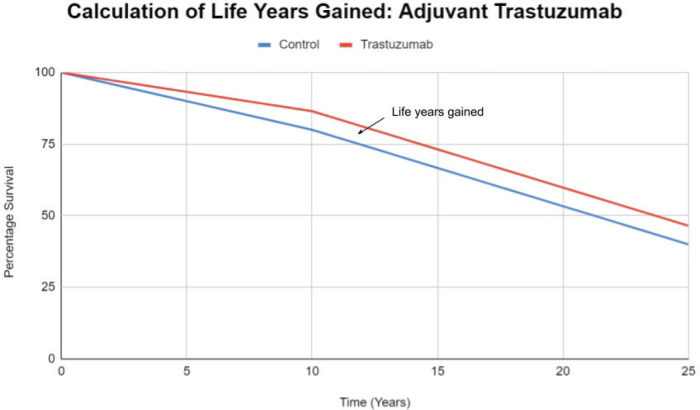
Calculation of life years gained in the curative setting.

In the adjuvant curative setting, we considered OS gains, where this was reported. In indications, where OS data was not mature we substituted DFS gain as the outcome measure. Although DFS gains may not directly translate into improvements in OS, we felt it was still important to perform an analysis to have some indication of cost-effectiveness. Life years gained in the curative setting were calculated as described fully in Supplementary Figure S1 and Supplementary Box 1. In summary, this calculation was performed assuming that on average the survivors at the end of follow-up would go on to live up to life expectancy.

The cost of each individual drug procured during the year 2021 was obtained from the Medical Supplies division of the Ministry of Health published on its website [6]. Since the procurement of drugs by the Ministry is done through an open tender procedure the price listed on the Ministry website is indicative of its true cost, there were no confidential discounts or rebates. This was converted to United States Dollars based on the average exchange rate for the year 2021.

The direct drug cost for a standard course of treatment was computed as for an adult male weighing 50 kg with a body surface area of 1.3. Indirect costs as well as costs of administration such as intravenous cannulas, infusion sets and so on, were not considered.

If the novel therapeutic agent was not an additive treatment, the costs of the drugs used in the comparator arm were subtracted from the cost of the novel agents.

The cost of treatment per patient was computed by multiplying the total dose required for a course of treatment by the unit cost. In curative settings, treatment is predetermined by protocol and the total drug dose for the whole course is computed.

For palliative indications, the median duration of treatment and/or number of treatment cycles were obtained from published studies and total drug dose was computed. If the median duration of treatment or treatment exposure was not reported the median progression-free survival was substituted since in the palliative setting treatment is often continued until disease progression.

### Cost per life year gained

The cost per life year gained was then computed by dividing the total cost of treatment by the life years gained by the treatment.

### Trials of treatment de-escalation

We also considered studies of statistically proven equivalence or non-inferiority of de-escalated treatment. We assumed that the survival gains of the de-escalated treatment were the same as a standard treatment and the cost of de-escalated treatment was computed and divided by the life-years gained of the standard treatment to determine the cost per life year gained by the de-escalated treatment.

### Cost-effectiveness thresholds

Three thresholds based on World Health Organisation recommendations were used for this analysis - viz: less than the per capita annual gross domestic product (GDP) per life year gained (GDP × 1 = US$3815; highly cost-effective), 3 times the per-capita annual GDP per life year gained (GDP × 3 = US$11445; cost-effective) and 4 times the per-capita annual GDP per life year gained (GDP × 4 = US$15260; potentially cost-effective with price reduction) were considered. The per capita GDP of Sri Lanka for the year 2021 was obtained to compute these thresholds [12,13].

### Total annual cost for each indication

The total cost of treatment per year for each indication for the entire country was computed by multiplying the total cost per patient by the estimated number of patients likely to be treated in each indication. While national incidence data for each cancer is available in the National Cancer Registry, there is a paucity of data on stage distribution. As such, three oncologists independently estimated the number of patients likely to be treated in each indication during a year. The mean value of the number estimated by the three oncologists was taken for the analysis.

The list of references used to obtain data for each treatment are listed in the Supplementary Table S1.

## Results

The average exchange rate for the year 2021 was 200 Sri Lankan Rupees per US$[7]. The per capita GDP for the year 2021 was US$ 3815. The cost of treatment per standard treatment course was obtained for 83 oncology drugs [8]. Conventional agents that were excluded from the analysis are shown in Supplementary Table S2 along with the cost per standard treatment course.

After the exclusion of these drugs, 41 drugs spanning across 83 indications in the palliative setting and 6 drugs across 10 indications in the adjuvant setting were included in the analysis. A full description of the analysis including the clinical trials from which outcome data was obtained from is included in the supplementary appendix.

There were two drugs for which de-escalated treatment was of relevance. When analysing abiraterone, we considered a low-dose treatment regimen of 250 mg with food and the standard dose of 1,000 mg on an empty stomach for each indication. Similarly for adjuvant trastuzumab we considered 6 months of treatment as well as 12 months of treatment.

**Figure 1.**
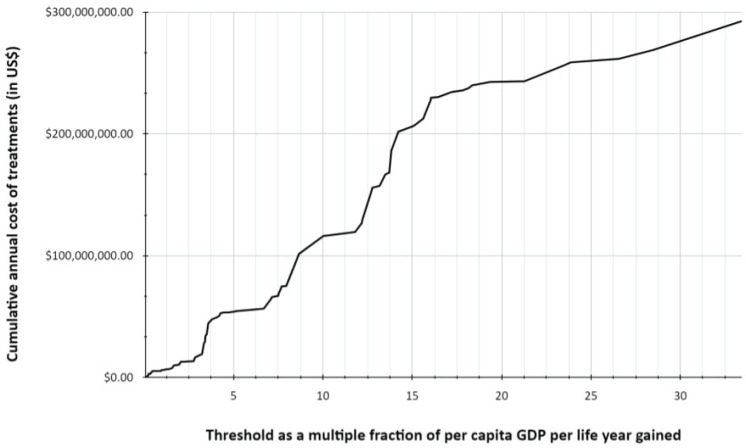
Cumulative cost of treatments of novel drugs in relation to cost-effectiveness thresholds based on per capita GDP per life year gained.

Tables 1 and 2 list the highly cost-effective and cost-effective drug indications, respectively, along with the total annual cost of procurement for these drugs. Supplementary Tables S3 and S4 list the drugs and indications that are potentially cost-effective and not cost-effective, respectively. Figure 1 shows the plot for cumulative annual procurement cost against per capita GDP per life year gained.

The total cost of treatment is US$ 6 million if a threshold of per capita GDP per life year gained GDP × 1 = US$3815 was set and US$ 13.2 million if it was per capita GDP × 3 = US$11445 per life year gained. If the threshold is increased to GDP × 4 = US$15260, the total cost would rise to US$ 47.3 million. If no threshold was set, Sri Lanka’s health system would need US$ 295 million to fund these novel drugs.

## Discussion

The allocation of funds for healthcare in a public funded state health system is determined by political authorities and is often influenced by the general macroeconomic situation of the country [9]. Once the allocation is decided, it is imperative that a budget impact analysis be performed and cost effectiveness-based thresholds be considered to ensure maximum benefit from the drugs procured by each health system [10]. In the absence of thresholds, frequent shortages of novel cancer therapeutics will be inevitable when the allotted budget is expended thereby denying these drugs to patients across all indications.

Through this study, we show that a simple budget impact analysis could provide some data for a cost-effectiveness threshold-based strategy to ensure sustainability in the provision of novel cancer therapeutics in health systems such as ours. We believe that our work would find resonance with healthcare systems of other low and middle-income countries (LMICs).

We used the per capita GDP-based thresholds as proposed by the World Health Organisation (WHO). There are a number of criticisms of setting cost-effectiveness thresholds based on per capita GDP. However, for health systems such as ours, the WHO thresholds provide a practical ‘starting-point’ [10,11,12]. In this respect our work provides evidence for the usefulness of the cut-off of per capita GDP × 3 = US$11445 per life year gained as the threshold for determining cost-effectiveness in LMICs [10,11,12].

Based on this threshold, the budget for procurement of novel cancer therapeutics would be approximately US$ 13.2 million which would be around 9% of the total drug budget of the state health system. Increasing the threshold to GDP × 4 = US$15260 would nearly triple the amount of funding required, further validating the robustness of the WHO threshold of GDP × 3. Without cost thresholds, the cumulative annual cost of the currently procured novel drugs would be nearly US$ 300 million, assuming that every eligible patient would receive treatment. This is around 1.5 times the total annual budget of all drugs in the state health sector and is clearly unsustainable. In a time of financial crisis, the lower threshold of per capita GDP per life year gained (GDP × 1 = US$3815) can be used to prioritise procurement. As shown by our data, a total allocation of approximately US$ 6 million would be sufficient to ensure the supply of these highly cost-effective drugs.

Furthermore, defining a cost-effectiveness threshold would also provide an incentive for pharmaceutical suppliers to reduce the price of drugs thereby leading to cost savings. These savings could be channeled to more cost-effective treatment modalities such as radiotherapy and surgery. Indeed, studies have shown that the dearth of quality radiotherapy resources in Sri Lanka has adversely impacted on outcomes of potentially curative cancers [14,15]. Investing in screening and streamlining early detection pathways may also lead to significant improvements in survival [16,17].

Except for ibrutinib which is not registered in Sri Lanka, all treatments mentioned in the WHO essential drugs list were found to be cost-effective in our setting as well [18]. However, there were several other treatments that were not included in the WHO list that were found to be cost-effective in our study, which are listed in Box 1. Two such treatments, adjuvant osimertinib in resected in adenocarcinoma of the lung and adjuvant olaparib in early germline BRCA mutation-positive breast cancer, are very recent developments [19,20]. Nevertheless, this underscores the importance of performing local cost-effectiveness assessments to take into account cost variations in different health systems.

Another salient finding of our work is the cost-savings that can be achieved by using lower doses of abiraterone for metastatic prostate cancer and a shorter duration of adjuvant treatment with trastuzumab in early breast cancer, both of which have robust evidence in the form of non-inferiority randomised clinical trials [21,22].

For abiraterone, a lower dose of 250 mg with food was shown to have equal efficacy in terms of biochemical response in castration-resistant prostate cancer [21]. The clinical equipoise can be safely extrapolated to the hormone-sensitive phase of the disease as well and this is borne out by the inclusion of the lower dose option in the NCCN guidelines for both settings [23].

Box 1. **Cost-effective treatments not included in the World Health Organisation list of essential medicines.**

1. Abiraterone for hormone sensitive metastatic prostate cancer.
2. Pomalidomide in the treatment of relapsed/refractory multiple myeloma.
3. Topotecan in the second line treatment of advanced cervical cancer.
4. Cabazitaxel in the treatment of metastatic castration resistant prostate cancer (post-docetaxel).
5. Fulvestrant in the first and second line treatment of endocrine sensitive metastatic breast cancer.
6. Sunitinib in the first line treatment of metastatic renal cell carcinoma treatment of gastrointestinal stromal tumour (GIST).
7. Nab-paclitxael in the first line treatment of unresectable advanced pancreatic cancer.
8. Bevacizumab for platinum refractory advanced epithelial ovarian cancer.
9. Trastuzumab for HER2 positive metastatic gastric cancer.
10. Adjuvant osimertinib in resected high risk EGFR mutation locoregional adenocarcinoma of lung.
11. Adjuvant olaparib for germline BRCA mutation positive HER2 negative high risk early breast cancer.
12. Sunitinib for first line treatment of metastatic renal cell cancer.
13. Pazopanib for first line treatment of metastatic renal cell cancer.
14. Sunitinib for second line treatment of unresectable gastrointestinal stromal tumours.
15. Pazopanib for second line treatment of metastatic or unresectable soft tissue sarcoma.

Even though the landmark PERSEPHONE trial of more than 4,000 patients proved non-inferiority for 6 months of adjuvant trastuzumab with 12 months of treatment, the oncology community has been slow to adopt this partly due to concerns with its subgroup analysis showing superiority of 12 months of treatment in patients receiving concurrent trastuzumab with chemotherapy [24]. However, clinicians in LMICs such as ours would be well advised to opt for 6 months of adjuvant trastuzumab due to its substantial cost savings and the uncertain benefit of extending adjuvant treatment to 1 year, which if at all, is likely to be marginal. The newer anti HER-2 monoclonal antibody Pertuzumab was not even remotely cost-effective either in the adjuvant or metastatic setting. Trastuzumab emtansine has not been used in the state health sector and we were, therefore, unable to perform an analysis of cost-effectiveness. However, unless very substantial price reductions are made these agents are unlikely to be cost-effective in our setting.

With regard to multiple drugs for the same indication, we found that abiraterone was substantially more cost-effective than enzalutamide in metastatic prostatic cancer both in its hormone-sensitive and castration-resistant phases. Similarly, gefitinib and erlotinib were superior to osimertinib in the first-line treatment of metastatic adenocarcinoma of the lung, while pembrolizumab was more cost-effective than nivolumab in metastatic melanoma.

Despite gaining approval for multiple malignancies, the immunotherapeutic agents Pembrolizumab and Nivolumab failed to reach the cost threshold in almost all indications with the exception of metastatic melanoma, where it was potentially cost-effective.

It is unlikely that these drugs would be affordable in the health systems of LMICs such as ours in the near future. However, encouraging results from recent trials exploring the efficacy of lower doses of these agents. Provides some hope, and more studies in this space are an imperative need [25].

Analysis of agents used in the first-line treatment of Chronic Myeloid Leukaemia posed many issues since it takes the form of a chronic disease entity where treatment extends beyond 10 years, When considering imatinib in the first-line treatment of chronic myeloid leukemia (CML), its cost is cheaper than the comparator interferon-alpha and low dose cytarabine. Since the annual treatment cost per patient for imatinib was only 180 US$ we excluded it from this analysis since its cost-effectiveness is evident. Due to short follow-up in the clinical trials of first-line treatment of CML, with the novel tyrosine kinase agents nilotinib and dasatinib, it was not possible to determine the survival gain accurately [26]. However, the overall survival gain over imatinib is likely to be very modest in the first-line setting and these agents are substantially more expensive than imatinib [26]. Over a 10-year period, nilotinib and dasatinib would cost US$ 53,000 and 44,347 more than imatinib, respectively.

We lacked the resources and expertise for a comprehensive health economic analysis as done by institutions such as the National Institute for Health and Care Excellence in the United Kingdom. In our analysis, we only considered the direct cost of the drug. The cost of drug administration, investigations, staff costs and so on, were not included. Furthermore, in the absence of data on quality of life in our setting, we were compelled to consider life years gained as the outcome variable rather than quality-adjusted life years. This also meant that more robust cost-effectiveness analysis methods such as a Markov model could not be performed. Since the WHO thresholds are based on quality-adjusted life years gains and not life years gained our data is likely to overestimate the benefit of treatment. More studies evaluating quality of life and cost of treatment in the state health sector are required as a matter of urgency.

The use of median overall survival gain may somewhat underestimate the benefit of immunotherapeutic agents where there is a ‘long-tail’ in the survival curve. We used mean restricted survival gain which is a more robust parameter, if this was reported in the publications.

Since survival gains in real-world populations could be significantly lower than that of patients treated in clinical trials, our analysis may have overestimated the benefit of novel cancer therapeutics.

For the budget impact analysis, we had to estimate the total number of cases per year for the whole country for each indication. While data on the incidence of each cancer is available in the National Cancer Registry, there is a paucity of data on other variables such as stage distribution, lines of treatment and so on. As such, the likely number of cases for each indication for the whole country was determined by taking the mean value of the estimates made by three oncologists. Sri Lanka has a centralised health system, and oncologists serve on rotation in centres throughout the country and we felt that a reasonable estimate could be obtain by this method.

Another potential drawback of our approach is that it does not factor in drugs repurposed for use in orphan diseases, since there is unlikely to be data from randomised trials in these settings. As the number of cases are low, the budgetary impact of using these repurposed drugs is likely to be minimal. The Sri Lankan public health system already has a mechanism in place for such drugs to be requested on a case-by-case basis.

We also did not consider the use of non-pharmacological alternatives such as orchiectomy for androgen deprivation in prostate cancer. The inability to determine quality-adjusted life year gains for novel therapeutics should not deter health systems such as ours from using more simple metrics when setting cost-effectiveness thresholds. As shown by our results even rudimentary analysis could provide valuable insights when making decisions on funding. Some data are certainly better than none in this regard.

Our intention was to highlight the importance of cost-effectiveness thresholds in determining novel cancer drug procurement and usage in health systems such as ours. We believe this has been achieved by our work, notwithstanding the limitations mentioned above.

## Conflicts of interest

None.

## Funding

This work did not receive any specific funding from any private, governmental or non-governmental institution.

## Ethics approval and consent to participate

Not applicable.

## Consent for publication

Not applicable.

## Figures and Tables

**Table 1.** Highly cost-effective treatments (Cost per life year gained less than per capita GDP).

Drug	Setting	Incidence of grade 3 or higher toxicity	ESMO magnitude of benefit scale	Survival gain	Cost per standard treatment course in US$	Cost per life year gained in US$	Cost per life year gained as fraction of per capita GDP	Estimated number of treated patients per year	Estimated total cost per year US$
Curative setting*									
Trastuzumab (6 months)	Adjuvant treatment of Non-metastatic breast cancer	3%	A	6.5 % (10 years OS)	1,502	1,156	0.3	880	1,322,000
Trastuzumab (12 months)	Adjuvant treatment of non-metastatic breast cancer	8%		6.5% (10 years OS)	2,951	2,270	0.6	880 m	2,597,161
Palliative setting**									
Rituximab (Maintenance)	Follicular lymphoma	10%	Not scored	79 months (PFS)	1,550	795	0.06	50	76,748
Bortezomib	Multiple Myeloma (First Line)	30%	Not scored	13.3 months	485	437	0.11	475	230,216
Abiraterone 250 mg	Metastatic hormone sensitive prostate cancer	20%	4	16.8 months	945	675	0.18	500	472,447
Abiraterone 250 mg	Metastatic castration resistant prostate cancer (Post-docetaxel)	20%	4	3.9 months	289	1,198	0.23	100	28,943
Rituximab (with chemotherapy)	Non-Hodgkin’s Lymphoma***	10%	Not scored	13 months	1,034	530	0.14	750	775,235
Gefitinib	Metastatic Adenocarcinoma of lung (first Line)	30%	4	5.4 months (PFS)	447	994	0.26	340	134,240
Abiraterone 250 mg	Metastatic castration resistant prostate Cancer (Pre-docetaxel)	20%	4	4.4 months	502	1,370	0.36	300	150,672
Erlotinib	Metastatic adenocarcinoma of lung (1st Line)	15%	4	8.5 months	1,003	2,314	0.37	340	340,902
Lenalidomide	Multiple Myeloma (Transplant Ineligible first line)	30%	Not Scored	13.2 months	1,704	1,550	0.41	475	809,629
Trastuzumab	Metastatic breast cancer	8%	Not assessed	4.8 months	2,146	1,764	0.46	264	566,653
Pomalidomide	Multiple Myeloma (second line)	15%	Not assessed	4.4 months	1,055	1,809	0.47	333	350,879
Abiraterone 1,000 mg	Metastatic hormone sensitive prostate cancer	20%	4	16.8 months	3,780	2,700	0.71	500	1,889,789
Abiraterone 1,000 mg	Metastatic castration resistant prostate cancer (Post docetaxel)	20%	4	3.9 months	1,158	3,562	0.93	100	115,771
Cabazitaxel	Metastatic castration resistant prostate cancer (Post docetaxel)	50%	3	2.6 months	774	3,573	0.94	100	77,423
Fulvestrant 250	Metastatic breast cancer (first line) in combination with anastrozole	20%	Not assessed	7.8 months	2,433	3,743	0.98	330	802,938
Total cost (using the most cost-effective option for each indication)									6,004,878.24

ESMO, European Society for Medical Oncology. PFS, Progression Free Survival. OS, Overall Survival

* Survival gain expressed as percentage gain in 10-year overall survival

** Survival gain expressed as gains is median/mean overall survival or progression-free survival

*** Survival gains are similar for both high-grade and indolent non-Hodgkin’s lymphoma and, therefore, a single analysis was performed

**Table 2.** Cost-effective treatments (drug cost per life year gained between 1 and 3 times per capita GDP).

Drug	Setting	ESMO magnitude of benefit score	Survival gain	Cost per standard treatment course in US$	Cost per life year gained in US$	Cost per life year gained as fraction of per capita GDP	Estimated number of treated patients per year	Estimated total cost per year in US$
Curative setting*								
Osimertinib	Adjuvant treatment of surgically resected stage IIB-III EGFR mutant adenocarcinoma of lung	A	50 %(3 years DFS)	29,692	6,252	1.64	55	1,633,036
Goserelin	Adjuvant treatment of localised prostate cancer treated with radical radiotherapy	Not scored	4.8 %(10 years DSS)	1,536	6,399	1.68	300	460,764
Olaparib	Adjuvant treatment of germline BRCA mutant early breast cancer	A	8.8%(3 years DFS)	21,746	7,845	2.06	110	2,392,052
Palliative setting**								
Topotecan	Metastatic cervical cancer (second line)	Not scored	2.9 months	1,015	4,201	1.1	110	111,672
Sunitinib	Metastatic renal cell carcinoma	4	14 months	5,619	4,816	1.26	100	561,900
Sunitinib	Advanced gastrointestinal stromal tumour (second line)	3	20.9 months	504,992	5,268	1.38	10	35,120
Trastuzumab	Metastatic adenocarcinoma of stomach	3	2.7 months	1,222	5,433	1.42	65	79,454
Abiraterone 1,000 mg	Metastatic castration resistant prostate cancer ( Pre docetaxel)	4	4.4 months	289	5,479	1.44	300	602,689
Fulvestrant 500 mg	Metastatic breast cancer (Second Line)	Not assessed	4.1 months	502	5,997	1.57	440	901,545
Pazopanib	Metastatic renal cell carcinoma	4	14 months	7,780	6,669	1.75	100	778,000
Bevacizumab	Platinum refractory advanced epithelial ovarian cancer	4	3.3 months	2,046	7,442	1.95	285	583,247
Everolimus	Advanced renal cell carcinoma (second line)	3	3 months	1,003	8,814	2.31	40	88,143
Nab-Paclitaxel	Unresectable pancreatic cancer	2	2.4 months	2,180	10,063	2.64	135	294,337
Pazopanib	Metastatic soft tissue sarcoma (second line)	3	3 months	2,640	10,559	2.77	26	68,634
Total cost (using the most cost-effective option for each indication)								7,209,904

DFS, Disease-Free Survival. ESMO, European Society for Medical Oncology

* Survival gain expressed as percentage disease-free DFS gain

** Survival gain expressed as gains is median/mean overall survival or progression-free survival

## Supplementary materials

Supplementary Box S1. Example calculation of life years gained in the adjuvant setting using trastuzumab in early breast cancer as an example. Overall survival gain at end of 10 years follow up = 6.5 % Assuming OS gain is proportionate throughout follow-up mean survival gain during follow-up=6.5%/2 = 3.25% Calculating Life years gained during follow-up: (OS gain as a fraction/2) * (duration of follow-up/2) (0.065/2) * 10 = 0.325 years Calculating Life years gained after follow-up Life years after follow up= (Average life expectancy- Median age at enrollment - Follow Up time) × OS gain as a fraction. Average life expectancy = 75 years Median age at enrolment = 50 years Duration of follow-up = 10 years (75 - 50 - 10 ) * 0.065 = 15 years * 0.065 = 0.975 years Calculating Total life years gained Life years gained during follow-up + Life years gained after follow-up 0.325 + 0.91 years = 1.3 years

**Supplementary Table S1.** List of references used for computation of cost-effectiveness of each treatment.

Drug	Indication	References
Rituximab	Follicular lymphoma maintenance	J Clin Oncol. 2019 Nov 1;37(31):2815–2824.
Gefinib	Metastatic non-small cell lung cancer first line EGFR mutation poitive	N Engl J Med 2010; 362:2380–2388
Erlotinib	Metastatic non-small cell lung cancer first line	Lancet Oncol 2011; 12: 735–42
Everolimus	Advanced renal cell carcinoma second line (Versus placebo)	Cancer 2010 Sep 15;116(18):4256–65
Pazopanib	Advanced unresectable sarcoma second line	Lancet. 2012;379(9829):1879
Palbociclib	Metastatic hormone sensitive breast cancer second line	N Engl J Med 2018; 379:1926–1936, N Engl J Med. 2015 Jul 16;373(3):209–19
Lapatinib	Advanced HER2+ breast cancer second line treatment	N Engl J Med 2006; 355:2733–2743
Palbociclib	Metastatic hormone sensitive breast cancer first line	N Engl J Med 2016; 375:1925–1936
Trabectidine	Advanced unresectable sarcoma second line	J Clin Oncol. 2016;34(8):786
topotecan	Advanced platinum refractory epithelial ovarian cancer	J Clin Oncol. 1997; 15(6):2183–2193
Ceritinib	Metastatic metastatic non-small cell lung cancer ALK+ first line	Lancet. 2017;389(10072):917.
Bevacizumab	Metastatic colorectal cancer first line	J Clin Oncol. 2008 Apr 20;26(12):2013–9
Crizotinib	Metastatic metastatic non-small cell lung cancer ALK+ first line (versus chemo)	N Engl J Med 2013; 368:2385–2394
Olaparib	Advanced platinum sensitive epithelial ovarian cancer	J Clin Oncol. 2020;38(11):1164
Nivolumab	Muscle invasive bladder cancer post cystectomy adjuvant treatment	N Engl J Med 2021; 384:2102–2114
Olaparib	Germline BRCA mutant and HER2-negative metastatic breast cancer	Ann Oncol. 2019;30(4):558
Vandatenib	Advanced unresectable medullary carcinoma of thyroid	J Clin Oncol. 2012 Jan 10;30(2):134–41
Nivolumab	Metastatic melanoma	J Clin Oncol. 2020 Nov 20;38(33):3937–3946
Pembrolizumab	Metastatic melanoma	Lancet Oncol. 2019 Sep;20(9):1239–1251
Sunitinib	Unresectable gastrointestinal stromal tumour second line	Lancet. 2006;368(9544):1329, Clin Cancer Res. 2012 Jun 1; 18(11): 3170–3179.
Rituximab	Non-Hodgkin's lympma first line	N Engl J Med 2002; 346:235-242 Blood. 2014; 124(21):1752 and J Natl Cancer Inst. 2007 May 2;99(9):706–14
Pembrolizumab	Metastatic non-small cell lung cancer PD1> 50% single agent first line treatment	J Clin Oncol. 2019 Mar 1;37(7):537–546
Olaparib	BRCA mutant epithelial ovarian cancer first line maintenance treatment	Lancet Oncol 2021;22(12):1721–1731
Pomalidamide	Multiple myeloma second line	Br J Haematol. 2015 Mar;168(6):820–3.
Trastuzumab	HER2+ metastatic breast cancer	J Clin. Oncol. 2005 Jul 1;23(19):4265–74.
Nivolumab	Squamous cell carcinoma of the head and neck second line	Oral Oncol. 2018 Jun;81:45–5
Sunitinib	Advanced renal cell carcinoma first line	J Clin Oncol. 2009;27(22):3584–3590.
Pazopanib	Advanced renal cell carcinoma first line	N Engl J Med 2013;369:722–31.
Cetuximab	Treatment refractory RAS wildtype metastatic colorectal cancer (as monotherapy)	N Engl J Med 2008; 359:1757–1765
Pembrolizumab	Unresectable PDL1+ squamous cell carcinoma of head and neck first line single agent treatment	Lancet. 2019 Nov 23;394(10212):1915–1928
Pembrolizumab	PDL1+ metastatic non-small cell lung cancer second line single agent	Lancet 2016; 387: 1540–50
Pembrolizumab	Metastatic non-small cell lung cancer (Non-squamous) first line in combination with chemotherapy	J Clin Oncol 2020 May 10;38(14):1505–1517
Panitumumab	Metastatic RAS wildtype colorectal cancer first line treatment in combination with FOLFOX	N Engl J Med 2013 Sep 12;369(11):1023–34.
Nivolumab	Adjuvant treatment in resected oesophageal or gastro-oesophageal juntional cancer	N Engl J Med 2021; 384:1191–1203
Pembrolizumab	Metastatic non-small cell lung cancer (Squmao)us first line in combination with chemotherapy	J Thoracic Oncol. 2020 Oct;15(10):1657–1669.
Bortezomib	Multiple Myeloma (trasnplant inlegible)	Blood. 2011;118(21):476
Pembrolizumab	Unresectable PDL1+ squamous cell carcinoma of head and neck first line treatment in combination with chemotherapy	Lancet. 2019 Nov 23;394(10212):1915–1928
Nivolumab	Metastatic non-small cell lung cancer second line	J Clin Oncol. 2021 Mar 1;39(7):723–733
Nivolumab	Advanced renal cell carcinoma second line	N Engl J Med 2015; 373:1803–1813
Nivolumab	Metastatic squamous cell carcinoma of oesophagus second line	Lancet Oncol. 2019 Nov;20(11):1506–1517.
Pertuzumab	Metastatic HER2+ breast cancer	N Engl J Med 2015; 372:724–734
topotecan	Metastatic cervical cancer second line	J Clin Oncol. 2005 Jul 20;23(21):4626–33
Pembrolizumab	PD1+ metastatic cervical cancer first line treatment in combination with chemotherapy	N Engl J Med 2021; 385:1856–1867
Lenalidomide	Multiple myeloma (trasnplant inlegible)	Blood 2018 Jan 18;131(3):301–310
Bevacizumab	Platinum refractory epithelial ovarian cancer	J Clin Oncol. 2014;32(13):1302–8
Panitumumab	Treatment refractory RAS wildtype metastatic colorectal cancer (as monotherapy)	Br J Cancer. 2016;115:1206–1214
Pembrolizumab	Metastatic triple negative breast cancer PDL1 > 10%	N Engl J Med 2022; 387:217–226
Bevacizumab	Metastatic cervical cancer	N Engl J Med 2014; 370:734–743
Osimertinib	Metastatic EGFR mutant non-small cell lung cancer second line	Ann Oncol. 2020 Nov;31(11):1536-1544
Ribociclib	Metastatic hormone sensitive breast cancer second line	N Engl J Med 2020; 382:514–524
Nab-Paclitaxel	Advanced pancreatic cancer	N Engl J Med 2013; 369:1691–1703
Pembrolizumab	Metastatic oesophageal or gastro-oesophageal junctional cancer PD1>10% first line treatment in combination with chemotherapy	Lancet 2021; 398: 759–71
Abiraterone 250 mg	Metastatic hormone sensitive prostate cancer	N Engl J Med 2017; 377: 338–51 and N Engl J Med. 2017;377:352-360
Abiraterone 1000 mg	Metastatic hormone sensitive prostate cancer	Lancet Oncol. 2019;20(5):686–700
Pembrolizumab	Metastatic oesophageal or gastro-oesophageal junctional cancer PD1>10% first line treatment singe agent	J Clin Oncol. 2020 Dec 10;38(35):4138–4148
Cetuximab	Unresectable squamous cell carcinoma of head and neck first line treatment in combination with chemotherapy	N Engl J Med 2008; 359:1116–1127
Fulvestrant 500 mg	Metastatic hormone sensitive breast cancer second line	J Natl Cancer Inst. 2014 Jan;106(1):djt337
Ribociclib	Metastatic hormone sensitive breast cancer first line	N Engl J Med 2022; 386:942–950
Decitabine	Acute myeloid leukaemia	J Clin Oncol. 2012. 30(21):2670–7
Azacitadine	Acute myeloid leukaemia	Blood. 2015. 126(3):291–299.
Cetuximab	Metastatic RAS wildtype left sided colorectal cancer first line treatment in combination with chemotherapy	JAMA Oncol. 2017;3(2):194–20
Enzalutamide	Metastatic castration resistant prostate cancer post docetaxel	N Engl J Med 2012; 367:1187–1197
Olaparib	BRCA mutant epithelial ovarian cancer maintenance treatment post second line chemotherapy	Lancet Oncol. 2021;22(5):620
Sorafenib	Advanced hepatocellular carcionoma	N Engl J Med 2008; 359:378-390
Fulvestrant 250 mg	Metastatic hormone sensitive breast Cancer first line	N Engl J Med 2019; 380:1226–1234
Cabazitaxel	Metastatic castration resistant prostate cancer after docetaxel and novel anti-androgen	N Engl J Med 2019; 381:2506–2518
Lenvatinib	Radioiodine refractory differentiated thyroid cancer	Eur J Cancer. 2021 Apr;147:51–57
Regorafenib	Treatment refractory metastatic colorectal cancer	Lancet.2013;381(9863):303
Abiraterone 250 mg	Metastatic castration resistant prostate cancer post docetaxel	N Engl J Med. 2011;3 64(21):1995–2005
Abiraterone 1000 mg	Metastatic castration resistant prostate cancer post docetaxel	N Engl J Med. 2011;3 64(21):1995–2005
Nivolumab	PDL1+ metastatic gastro-eosphageal junctional or gasric cancer metastatic first line	Lancet 2021 Jul 3;398(10294):27–40
Trastuzumab	Metastatic HER2+ gastric cancer	Lancet 2010; 376:687–97
Bevacizumab	Metastatic colorectal cancer second line	J Clin Oncol. 2007;25(12):1539–44
Ribociclib	Metastatic hormonse sensitive breast Cancer (Pre-menopausal) first line	Clin Cancer Res. 2022 Mar 1;28(5):851–859
Bevacizumab	Platinum sensitive relapsed epithelial ovarian cancer	Lancet Oncol. 2017;18(6):779–791
Bevacizumab	Metastatic non-squmouas non-small cell lung cancer	N Engl J Med 2006; 355:2542–2550
Bevacizumab	Metastatic epithelial ovary cancer first line	Lancet Oncol 2015; 16:928–36
Osimertinib	Metastatic non-small cell lung cancer first line	N Engl J Med 2020; 382:41–50
Abiraterone 250 mg	Metastatic castration resistant prostate cancer pre docetaxel	Lancet Oncol. 2015 Feb;16(2):152–60.
Abiraterone 1000 mg	Metastatic castration resistant prostate cancer pre docetaxel	Lancet Oncol. 2015 Feb;16(2):152–60.
Nilotinib	Imatinib resistant chronic myeloid Leukaemia (in comparison to high dose imatinib)	Value Health. 2011 Dec;14(8):1057–67
Enzalutamide	Metastatic castration resistant prostate cancer pre docetaxel	Eur Urol 2017;71(2):151–4
Dasatinib	Imatinib resistant chronic myeloid leukaemia (in comparison to high dose imatinib)	Value Health. 2011 Dec;14(8):1057–67
Trastuzumab	Adjuvant treatment of HER2+ breast cancer 6 months	Lancet. 2019;393(10191):2599–2612
Trastuzumab	Adjuvant treatment of HER2+ breast cancer 12 months	Lancet Oncol. 2021;22(8):1139–1150
Goserelin	Adjuvant treatment of localised prostate cancer	J Clin Oncol 26: 2497–2504
Osimertinib	Adjuvant treatmend of resected stage IB-III EGFR mutant adenocarcinoma of lung	N Engl J Med 2020; 383:1711–1723; Ann Oncol. 2022;33(Suppl_7):S1413–S1414
Olaparib	BRCA mutant high risk resected early breast cancer	N Engl J Med 2021;384(25):2394
Pembrolizumab	Stage III resected melanoma	Lancet Oncol 2021; 22: 643–54
Nivolumab	Stage III resected melanoma	Eur J Cancer 2020 Jun;132:176–186
Pembrolizumab	Adjuvant treatment of triple negative early breast cancer	N Engl J Med 2022; 386:556–567
Pembrolizumab	Adjuvant treatment of resected Renal Cell Carcinoma	N Engl J Med 2021; 385:683–694
Pertuzumab	Adjuvant treatment of HER2+ Breast Cancer	N Engl J Med 2017; 377:122–13

**Supplementary Table S2.** Conventional agents excluded from the analysis and cost per treatment course.

Drug	Treatment description	Cost per course (In US$)
Anastrozole	1 mg daily for 5 years	106
Asparaginase	Total of 27 doses at 6,000 IU/m^2^	666
Bicalutamide	50 mg daily for 2 years	121
Bleomycin	10 doses of 30,000 IU	251
Calcium Folinate	12 cycles of 400 mg /m^2^ per cycle	125
Capecitabine	8 cycles of 1,000 mg / m^2^ twice daily for 14 days per cycle	142
Carboplatin	6 cycles of 6 times area under the curve (Assumed 600 mg per cycle)	165
Chlorambucil	10 mg/m^2^/day days 1–7 per cycle for 12 cycles	944
Cisplatin	4 cycles of 25mg/m^2^ days 1–5 per cycle (BEP)	34
Cladribine	Single course of 0.1 mg/ kg days 1–7	676
Cyclophosphamide	8 cycles of 750 mg / m^2^ per cycle (CHOP)	20
Cytarabine	2 cycles of high dose cytarabine3 mg / m^2^ twice daily days 1, 3 and 5 per cycle	551
Dacarbazine	6 cycles of 375 mg / m^2^ days 1 and 15 per cycle (ABVD)	174
Dactinomycin	8 cycles of 0.75 mg / m^2^ days 1 and 2 per cycle (VAI)	509
Daunorubicin	3 cycles of 60 mg / m^2^ days 1–3 per cycle	115
Doxorubicin	8 cycles of 50 mg/m^2^ per cycle (CHOP)	46
Exemestane	25 mg daily for 5 years	330
Epirubicin	6 cycles of 100 mg/m^2^ per cycle (FEC-100)	120
Etoposide	4 cycles of 100 mg/m^2^ days 1–5 per cycle (BEP)	101
5-Fluorouracil	12 cycles of 400 mg /m^2^ loading dose and 2,400 mg / m^2^ infusion per cycle	112
Gemcitabine	6 cycles of 1,000 mg /m^2^ days 1 and 8 per cycle	77
Hydroxyurea	500 mg daily for 2 years	290
Ifosfamide	4 cycles of 1,200 mg / m^2^ days 1–5 per cycle (VIP – germ cell tumours)	279
Imatinib	400 mg daily	180
Irinotecan	12 cycles of 400 mg / m^2^ per cycle	452
Letrozole	2.5 mg daily for 5 years	62
Melphalan (Intravenous)	Single dose of 140 mg/m^2^	312
Melphalan (Oral)	12 cycles of 0.25 mg/kg days 1–4 per cycle	20
Methotrexate Oral	30 mg / m^2^ weekly for 3 years (maintenance treatment of acute lymphoblastic leukaemia/lymphoma)	25
Intravenous Methotrexate (High Dose)	4 cycles of 12 mg / m^2^ per cycle	696
Mercaptopurine	150 mg daily for 3 years (maintenance treatment of acute lymphoblastic leukaemia/lymphoma)	161
Oxaliplatin	8 cycles of 130 mg / m^2^ per cycle	94
Paclitaxel	8 cycles of 175 mg / m^2^ per cycle	97
Procarbazine	4 cycles of 100 mg/m^2^ days 1–10 per cycle	254
Tamoxifen	20 mg daily for 10 years	92
Temozolomide	6 weeks of 75 mg/m^2^ daily with radiotherapy and 6 cycles of 200 mg/m^2^ days 1–5 per cycle	40
Thalidomide	Maintenance treatment in multiple myeloma 100 mg for 1 year	46
Vinblastine	ABVD – 6 cycles of 6 mg/m^2^ day 1 and 15	53
Vincristine	CHOP – 8 cycles of 2 mg	21

**Supplementary Table S3.** Potentially cost-effective treatments (Drug cost per life year gained between 3-4 times per capita GDP).

Drug	Setting	Survival gain(ESMO magnitude of benefit score in parenthesis)	Cost per standard treatment course in US$	Cost per life year gained in US$	Cost per life year gained as Fraction of per capita GDP	Estimated number of treated patients per year	Estimated total cost per year in US$
Curative setting*							
Pembrolizumab	Adjuvant treatment of surgically resected stage III melanoma	18.4 % DFS (A)	59,799	13,684	3.59	100	5,979,899
Palliative setting***							
Olaparib	Maintenance treatment of newly diagnosed BRCA mutant Epithelial Ovarian Cancer	42.2 months (4)	43,492	12,367	3.24	67	2,892.209
Ribociclib	Metastatic hormone sensitive breast cancer (first line)	12.2 months (4)	13,296	12,765	3.35	440	8,775,678
Sorafenib	Advanced hepatocellular cancer	2.8 months (3)	3,010	12,898	3.38	250	752,412
Bevacizumab	Metastatic colorectal cancer (second line)	2.1 months (3)	2,274	12,994	3.41	250	568,467
Nivolumab	Metastatic melanoma	26.1 months (4)	28,342	13,031	3.42	165	4,676,382
Bevacizumab	Advanced ovarian cancer (second line treatment of platinum sensitive relapse)	4.9 months	5,457	13,365	3.5	285	1,555,327
Osimertinib	EGFR mutant adenocarcinoma of lung (second line)	10 months (4)	11,199	13,438	3.52	255	2,855,677
Ribociclib	Metastatic ER+ breast cancer (second line)	7.7 months	8,864	13,815	3.62	440	3,900,302
Bevacizumab	Stage IV ovarian epithelial carcinoma	4.8 months	5,798	14,496	3.8	650	3,305.069
Total cost							35,268,337

DFS, Disease Free Survival. ESMO, European Society for Medical Oncology

* Survival gain expressed as percentage disease free DFS

*** Survival gain expressed as gains is median/mean overall survival or progression free survival

**Supplementary Table S4.** Treatments which are not cost-effective (Cost per life year gained more than 4 times per capita GDP).

Drug	Indication	Cost per life year gained (in multiples of per capita GDP)	Estimated total cost per year
Palbociclib	Hormone sensitive metastatic breast cancer second line	4.03	$3,042,792
Bevacizumab	Metastatic cervical cancer	4.06	$1,575,791
Pembrolizumab	Metastatic melanoma	4.10	$4,062,814
Lapatinib	Metastatic HER2 positive breast cancer second line	4.19	$1,171,205
Decitabine	Acute myeloid leukaemia	4.21	$286,479
Palbociclib	Metastatic breast CA first line	4.22	$9,128,377
Nilotinib	Imatinib resistant chronic myeloid leukaemia	4.27	$1,980,848
Nivolumab	Adjuvant treatment of stage III melanoma	4.47	$11,053,266
Ribociclib	Pre-menopausal hormone sensitive metastatic breast cancer first line	4.47	$668,623
Trabectedin	Metastatic soft tissue sarcoma second line	4.75	$105,986
Azacitidine	Acute myeloid leukaemia	5.08	$604,654
Regorafenib	Metastatic treatment refractory colorectal cancer	5.12	$284,925
topotecan	Platinum refractory advanced ovarian cancer	6.05	$1,205,545
Ceritinib	Metastatic ALK+ non-small cell lung cancer	6.69	$922,244
Osimertinib	EGFR mutation metastatic adenocarcinoma of lung first line	7.08	$7,462,746
Bevacizumab	Metastatic colorectal cancer first line	7.15	$1,989,636
Enzalutamide	Metastatic castration resistant prostate cancer post-docetaxel	7.50	$1,058,314
Bevacizumab	Metastatic non-squamous non-small cell lung cancer	7.51	$1,217,657
Pembrolizumab	Metastatic non-small cell lung cancer PD-L1>50% first line single agent use	7.70	$6,577,889
Crizotinib	Metastatic ALK+ Non-small cell lung cancer	7.95	$335,216
Pembrolizumab	Adjuvant treatment of triple negative breast cancer	8.66	$26,311,558
Olaparib	BRCA mutant ovarian cancer maintenance treatment	10.04	$14,823,277
Pembrolizumab	Unresectable squamous cell carcinoma of head and neck PD-L1 positive single agent use	10.54	$844,221
Dasatinib	Imatinib resistant chronic myeloid leukaemia	11.80	$2,359,852
Pembrolizumab adjuvant RCC	Adjuvant treatment of renal cell carcinoma	12.17	$7,175,879
Lenvatinib	Radioiodine refractory advanced thyroid cancer	12.22	$3,515,879
Pembrolizumab	Metastatic non-squamous non-small lung cancer in combination with chemotherapy (first line use)	12.73	$23,321,608
Cetuximab	Metastatic treatment refractory colorectal cancer RAS wild type single agent use	12.77	$2,384,422
Pembrolizumab	Metastatic PD-L1 positive non-small cell lung cancer (second line single agent use)	13.17	$1,494,975
Panitumumab	Metastatic colorectal cancer RAS wild type first line use with chemotherapy	13.49	$9,327,889
Nivolumab	Unresectable squamous cell carcinoma of head and neck second line	13.72	$1,700,503
Pembrolizumab	Metastatic squamous cell carcinoma of lung first line use in combination with chemotherapy	13.83	$17,939,698
Pertuzumab	Metastatic HER2 positive breast cancer first line	14.22	$15,619,503
Pembrolizumab	Unrecetable PD-L1 positive squamous cell carcinoma of head and neck in combination with chemotherapy	15.00	$4,221,106
Olaparib	BRCA mutant platinum sensitive ovarian cancer single agent use (third line)	15.13	$959,353
Enzalutamide	Metastatic castration resistant prostate cancer pre-docetaxel	15.62	$5,507,162
Pembrolizumab	Metastatic triple negative breast cancer PD-L1 10% or more	16.04	$15,477,387
Nivolumab	Adjuvant treatment of muscle invasive bladder cancer	16.05	$1,657,990
Olaparib	BRCA mutant metastatic breast cancer second line treatment	16.46	$644,871
Nivolumab	Adjuvant treatment of oesophageal or gastroesophageal junctional adenocarcinoma	17.20	$4,052,864
Nivolumab	Metastatic squamous cell carcinoma of oesophagus (second line)	17.83	$1,417,085
Nivolumab	Metastatic non-small cell lung cancer (second line)	17.83	$1,445,427
Nivolumab	Metastatic renal cell carcinoma (second line)	18.16	$1,870,553
Panitumumab	Metastatic treatment refractory colorectal cancer RAS wild type single agent use	18.36	$2,261,307
Pembrolizumab	Metastatic PD-L1 positive cervical cancer in combination with chemotherapy (first line)	19.36	$2,708,543
Pembrolizumab	Metastatic adenocarcinoma of oesophagus or gastroesophageal junction PD-L1 > 10% single agent (second line)	21.28	$571,608
Cetuximab	Metastatic left sided colorectal cancer RAS wild type in combination with chemotherapy	23.48	$13,065,327
Pembrolizumab	Metastatic adenocarcinoma of oesophagus or gastroesophageal junction PD-L1 > 10% in combination with chemotherapy (first line)	23.86	$2,515,075
Vandatenib	Metastatic medullary carcinoma of thyroid	26.54	$2,834,715
Cetuximab	Unresectable squamous cell carcinoma of head and neck in combination with chemotherapy (first line)	28.48	$7,335,075
Pertuzumab adjuvant breast	Adjuvant treatment of HER2 positive breast cancer	33.44	$23,741,644
Nivolumab	Metastatic PD-L1 positive adenocarcinoma of oesophagus or gastroesophageal junction in combination with chemotherapy (first line)	37.82	$2,579,095
Total cost per year			$248,151,479
